# A Rare Case of Pneumomediastinum and Subcutaneous Emphysema Secondary to Recreational Nitrous Oxide Inhalation

**DOI:** 10.7759/cureus.88331

**Published:** 2025-07-19

**Authors:** Nicole C Oparaugo, Jay Cadavona, Sean D Delshad

**Affiliations:** 1 Internal Medicine, University of California, Irvine, Irvine, USA; 2 Hospital Medicine, University of California, Los Angeles, Los Angeles, USA

**Keywords:** laughing gas, nitrous oxide, pneumomediastinum, recreational drug use, subcutaneous emphysema, substance use disorder (sud)

## Abstract

The recreational inhalation of nitrous oxide, also known as laughing gas, continues to rise due to its euphoric effects and easy accessibility. Clinicians may therefore encounter patients suffering from the potential adverse effects of nitrous oxide inhalation more commonly. We present a case of a 35-year-old man who presented with neck and facial swelling and pleuritic chest pain after a two-day recreational binge of nitrous oxide inhalation. The patient was found to have subcutaneous emphysema and pneumomediastinum, rare adverse effects of nitrous oxide inhalation. The patient’s symptoms improved with supportive care.

## Introduction

Nitrous oxide, also known as laughing gas, is a colorless gas that has been utilized for many years in dentistry and medicine for its anesthetic and anxiolytic properties [1]. There is also a long history of recreational use of nitrous oxide, given its euphoric effects, with significant increases in its abuse since 2002 [2]. The gas can be inhaled via balloons, tanks, or whippets, small highly pressurized canisters that were traditionally used for whipped cream dispensers and are easily accessible [3]. The recreational use of nitrous oxide has been associated with various adverse effects, including, albeit rarely, pneumomediastinum and subcutaneous emphysema [4-8].

Pneumomediastinum is the presence of air in the chest between the lungs, which can develop after trauma, increased pressure in the chest, or various other situations in which air can leak from the lungs, airways, or gastrointestinal tract into the chest cavity [9]. Nitrous oxide inhalation can cause high alveolar pressure and rupture, resulting in pneumomediastinum [4]. Subcutaneous emphysema is the presence of air underneath the skin and is usually associated with the presence of air in deeper spaces, such as in pneumomediastinum [10]. Subcutaneous emphysema and pneumomediastinum are typically benign entities and self-resolve. However, large-volume pneumomediastinum may be life-threatening due to the compression of cardiopulmonary structures, and therefore, at least short-term close monitoring is recommended [11,12].

We present a case of a 35-year-old man who presented with facial and neck swelling after a binge of recreational nitrous oxide inhalation and was found to have pneumomediastinum and subcutaneous emphysema.

## Case presentation

**Figure 1:**
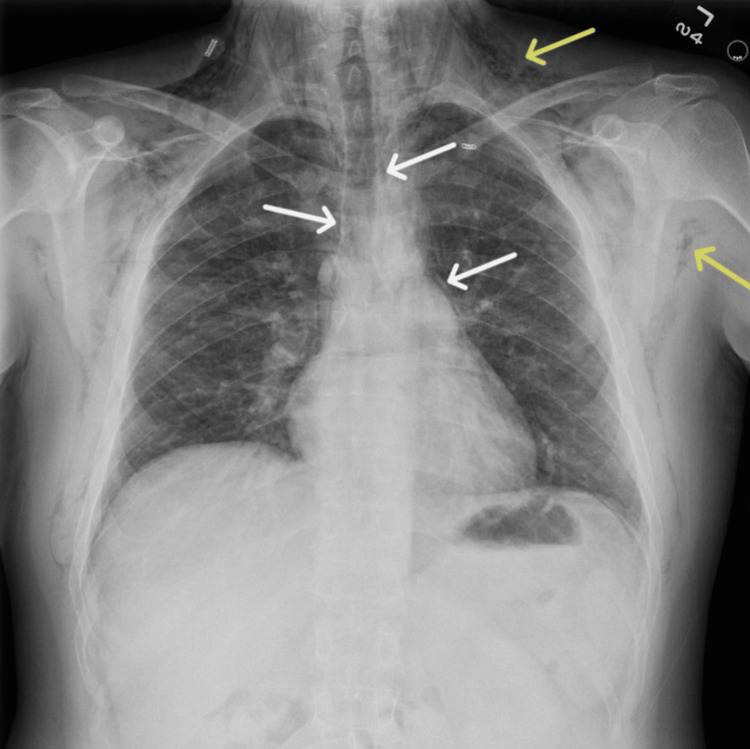
Posterior-anterior chest X-ray demonstrating pneumomediastinum (white arrows) and subcutaneous emphysema (yellow arrows).

**Figure 2:**
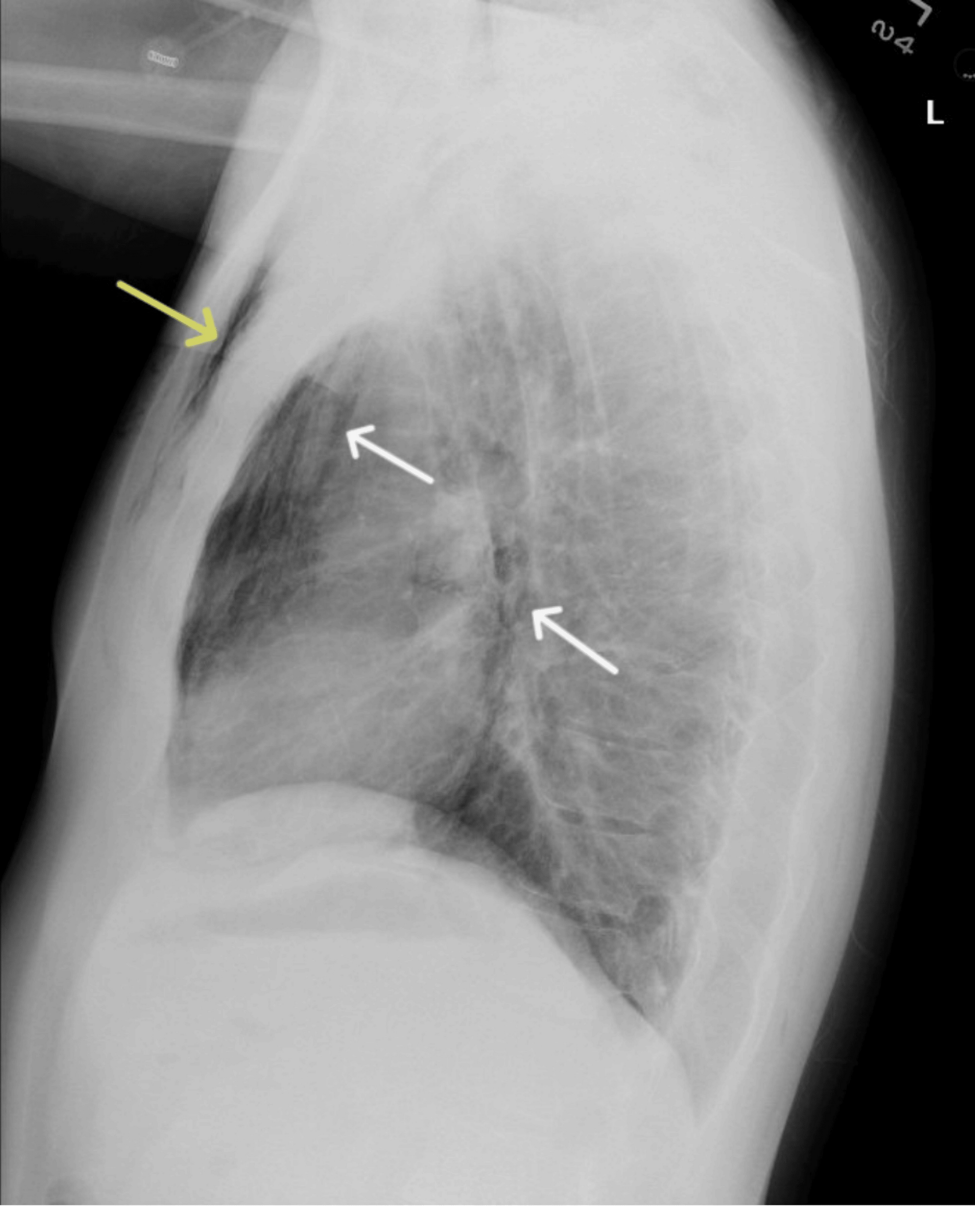
Lateral chest X-ray demonstrating pneumomediastinum (white arrows) and subcutaneous emphysema (yellow arrow).

A 35-year-old man with a history of anxiety, depression, and substance use disorder presented to the emergency room with one day of facial and neck swelling, pain with neck movement, pleuritic chest pain, odynophagia, and vomiting. The patient endorsed recreational inhalation of nitrous oxide directly from multiple tanks for two days prior to his presentation. The patient denied confusion, fever, paresthesias, or other substance use. Vital signs upon his presentation were a temperature of 37.1 degrees Celsius, blood pressure of 126/93 mmHg, heart rate of 133 beats per minute, respiratory rate of 18 breaths per minute, and an oxygen saturation of 97% at room air. Physical examination was notable for facial swelling and palpable crepitus of the neck and anterior chest below the clavicles bilaterally. Laboratory results were significant for a white blood cell count of 14,640/μL (normal: 4,160-9,950/μL) and a creatinine of 1.57 mg/dL (normal: 0.6-1.3mg/dL). A urine toxicology screen was negative. The electrocardiogram showed sinus tachycardia. Chest radiography demonstrated pneumomediastinum and extensive chest wall subcutaneous emphysema (Figures 1, 2).

The patient was admitted for monitoring, including continuous pulse oximetry, and started on supplemental oxygen via nasal cannula. The patient was kept nil per os and treated with intravenous fluids for acute kidney injury (AKI). A gastrografin esophagram demonstrated no esophageal perforation, and the patient’s diet was advanced as tolerated. On hospital day 1, the patient had normal vital signs, and repeat laboratories demonstrated a normal white blood cell count and the resolution of the AKI. A repeat chest radiograph demonstrated a stable small pneumomediastinum and was negative for other findings. The patient was monitored on continuous pulse oximetry, which remained normal during the hospitalization. The patient had progressive improvement in his symptoms and examination findings over the course of two days. The patient was ultimately discharged home with the recommendation to seek out substance use rehabilitation services. Unfortunately, the patient was lost to follow-up.

## Discussion

The presented patient developed extensive subcutaneous emphysema and pneumomediastinum following a two-day binge of inhaling nitrous oxide directly from the tank. The patient’s symptoms and findings improved with abstaining from further inhalation and supportive care. Given his history of vomiting, the differential diagnosis for his pneumomediastinum included esophageal perforation, and therefore, the patient underwent an esophagram to ensure no esophageal perforation was present prior to the safe resumption of his diet.

The patient’s AKI was most likely secondary to hypovolemia, given resolution with intravenous fluid resuscitation. Other toxic causes were unlikely given a negative urine toxicology screen. There is no strong evidence that the inhalation of nitrous oxide can cause AKI. However, inhaled nitrous oxide therapy has been shown to increase the risk of AKI in patients with acute respiratory distress syndrome. This may be due to nitrous oxide oxidative products that contribute to the apoptosis of glomerular, collecting duct, and distal convoluted tubular cells [13].

The patient initially had a leukocytosis that quickly resolved. The inhalation of nitrous oxide has not been previously associated with leukocytosis but rather leukopenia [14]. The patient’s leukocytosis was likely due to a stress reaction, given that there were no clear signs of infection and the patient showed rapid improvement with supportive care alone.

Recreational nitrous oxide use continues to become more prevalent. In a global survey, respondents aged 16-24 years old reporting recreational nitrous oxide use in the last 12 months increased from 10% in 2015 to 20% in 2021 [15]. As such, medical providers may see more patients presenting with adverse effects associated with its use. In 2022, the United Kingdom’s Advisory Council of the Misuse of Drugs demonstrated an increase in hospital admissions related to inhaled anesthetics [16].

More commonly known adverse effects of recreational nitrous oxide inhalation include pneumothorax and functional vitamin B12 deficiency [17,18]. Nitrous oxide inactivates vitamin B12, potentially resulting in neuropathy, ataxia, and macrocytic anemia [19]. The presented patient did not show signs of functional B12 deficiency, which typically occurs after more chronic use of inhaled nitrous oxide.

Pneumomediastinum and subcutaneous emphysema, as seen in the presented case, are more rare adverse effects that can occur after nitrous oxide inhalation [20]. Inhalation, especially directly from tanks or canisters, can lead to very high intra-alveolar pressure, causing alveolar rupture [4]. Free air is then able to track along the bronchovascular tree, resulting in pneumomediastinum and subcutaneous emphysema. With time and abstinence from further nitrous oxide inhalation, the free air ultimately resorbs, and symptoms typically resolve.

Other causes of pneumomediastinum include excessive coughing associated with exacerbations of chronic pulmonary disease, any action with forceful Valsalva such as childbirth, and traumatic injuries to the chest or abdomen. Pneumomediastinum and subcutaneous emphysema may be diagnosed on chest radiograph, as seen in Figure 1 and Figure 2, as linear or curvilinear lucencies outlining the mediastinal contours and striated lucencies in the soft tissues, respectively [21]. Pneumomediastinum typically self-resolves, and patients require short-term monitoring to ensure that there is no progression or ongoing air leak that can be life-threatening. Patients should be treated for any underlying pulmonary pathology and provided with antitussives and analgesics as needed. Supplemental oxygen may increase gas resorption and should be considered as supportive management [9].

## Conclusions

In conclusion, the recreational inhalation of nitrous oxide continues to rise, and clinicians should be wary of potential adverse effects, including functional vitamin B12 deficiency, pneumothorax, and pneumomediastinum. Pneumomediastinum resolves with the cessation of nitrous oxide use, but some level of monitoring and supportive care may be necessary for concomitant presenting signs and symptoms, such as leukocytosis or AKI. Given the rising recreational use of nitrous oxide and its associated adverse effects, further preventative measures, such as public health education, may be warranted.
